# Functional divergence of FTL9 and FTL10 in flowering control in rice

**DOI:** 10.1186/s12864-024-10441-9

**Published:** 2024-06-05

**Authors:** Jingai Tan, Sajid Muhammad, Lantian Zhang, Haohua He, Jianmin Bian

**Affiliations:** 1 Key Laboratory of Crop Physiology, Ecology and Genetic Breeding, Ministry of Education, Jiangxi Agricultural University, Nanchang, 330045 China; 2 Zhejiang Provincial Key Laboratory of Crop Genetic Resources, College of Agriculture and Biotechnology, Zhejiang University, Hangzhou, Zhejiang 310058 China

**Keywords:** Rice, Flowering time, *OsFTL9*, *OsFTL10*, Short-day

## Abstract

**Background:**

Floral transition in cereals is a critical phenomenon influenced by exogenous and endogenous signals, determining crop yield and reproduction. *Flowering Locus T-*like (*FT-*like) genes encode a mobile florigen, the main signaling molecule for flowering.

**Results:**

In this study, we characterized two *FT-*like genes, *FTL9* and *FTL10*, to study their functional diversity in flowering control in rice. We compared independent mutant lines of *ftl10* with WT and observed negligible differences in the flowering phenotype, or agronomic traits implying potentially redundant roles of *FTL10* loss-of-function in flowering control in rice. Nevertheless, we found that overexpression of *FTL10*, but not *FTL9*, substantially accelerated flowering, indicating the flowering-promoting role of *FTL10* and the divergent functions between *FTL9* and *FTL10* in flowering. Besides flowering, additive agronomic roles were observed for *FTL10-OE* regulating the number of effective panicles per plant, the number of primary branches per panicle, and spikelets per panicle without regulating seed size. Mechanistically, our Y2H and BiFC analyses demonstrate that *FTL10*, in contrast to *FTL9*, can interact with *FD1* and *GF14c*, forming a flowering activation complex and thereby regulating flowering.

**Conclusion:**

Altogether, our results elucidate the regulatory roles of *FTL9* and *FTL10* in flowering control, unveiling the molecular basis of functional divergence between *FTL10* and *FTL9*, which provides mechanistic insights into shaping the dynamics of flowering time regulation in rice.

**Supplementary Information:**

The online version contains supplementary material available at 10.1186/s12864-024-10441-9.

## Introduction

Flowering in plants involves transitioning from the vegetative to the reproductive phase to ensure reproduction. Several endogenous and exogenous cues are involved in flowering time regulation; however, appropriate time and day length are critical for flowering induction [1]. Flowering is timely induced by a flowering activation complex (FAC), organized by FLOWERING LOCUS T (FT), a small phosphatidylethanolamine binding protein (PEBP), and FD, a bZIP transcription factor with the assistance of scaffold protein 14-3-3 (GF14 in rice), when plants sense to favorable flowering environmental conditions [2]. In angiosperms, the *PEBP* gene family can be divided into three major clades: *MOTHER OF FT AND TFL1-*like (*MFT-*like), *TERMINAL FLOWER1-*like (*TFL1-*like), and *FT*, which are involved in regulating several growth and developmental processes, including seed germination, flowering time, and plant architecture [3]. Several *FT-*like genes have been identified in various plant genomes, including *O. sativa* (13 *FT-*like homologs), *Z. maize* (15 FT-like homologs), and *T. aestivum* and *barley* (12 *FT-*like homologs each), mostly involved in flowering time regulation [4–6].

Moreover, among the thirteen FTLs, two florigen genes, *Hd3a* and *RFT1*, regulate flowering in rice under short days (SDs) and long days (LDs), respectively [7]. Both *Hd3a* and *RFT1* are produced and expressed in the vascular tissues and transported to the shoot apical meristem (SAM) through the phloem. Suppression of *Hd3a* results in increased *RFT1* expression, potentially substituting *Hd3a* as a floral activator by delaying flowering [7]. Additionally, activation of *RFT1* in *Hd3a* RNAi plants correlates with higher levels of H3k9 acetylation at the *RFT1* locus, potentially enhancing *RFT1* expression [8, 9]. Interestingly, the knockdown of both *Hd3a* and *RFT1* completely blocked flowering in rice under SDs, depicting the necessary and overlapping functions of these two genes for flowering in rice [7]. The genome-wide analysis reveals the overlapping functions of *Hd3a* and *RFT1* in identifying a common set of targets crucial for floral transition as well as for other interconnected developmental processes in rice [10].

Recently, a study revealed that *Hd3a* and *RFT1* activated FLOWERING LOCUS T-LIKE 1 (FT-L1), a florigen-like protein with unique characteristics, collaborates with *Hd3a* and *RFT1* to facilitate vegetative-to-inflorescence meristem transition and regulate panicle branching by increasing determinacy in distal meristems [11]. Several positive and negative regulators control the expression of the florigens in leaves. Among them, the zinc finger transcription factor *Heading date 1* (*Hd1*), a homolog of Arabidopsis CONSTANS, promotes expression of *Hd3a* and *RFT1* under SD conditions, while it represses it under LD conditions [12, 13]. It was observed previously that *Hd3a*/*RFT1* interact with 14-3-3 proteins in the SAM of cytoplasm and enter the nucleus to combine with OsFD1, forming FAC that induces the expression of downstream flowering-related genes *OsMADS14* and *OsMADS15* [2].

However, RICE CENTRORADIALIS (RCN), rice TFL1-like proteins, compete with *Hd3a* for 14-3-3 binding and represses florigenic activity by forming florigen repression complex (FRC) with *OsFD1*. The balance between the FRC and FAC is regulated depending on the ratio of *Hd3a* to RCN in the cell. The FRC-FAC balance regulates SAM development, where under inductive SDs *Hd3a* accumulation in the SAM competes with RCN for FAC formation, initiating the reproductive transition while RCN knockdown reduces FRC formation allowing *Hd3a* to form FAC, ultimately hindering inflorescence development [2].

*Hd3a* and *RFT1* proteins can engage in both florigen activation and repression complexes [14]. Activation complexes to promote flowering depend on *OsFD1*. However, closely related additional bZIPs, including *Hd3a* BINDING REPRESSOR FACTOR1 (HBF1) and HBF2, form repressor complexes that reduce *Hd3a* and *RFT1* expression to delay flowering. The preference of *RFT1* and *Hd3a* to interact with *OsFD1* or the HBFs can be driven by relative expression patterns or modifications of *OsFD1* and the HBFs under different growing conditions [14].

Furthermore, different *FT-like* members can fine-tune heading date and plant architecture by regulating the balance of FAC and FRC in rice. For example, *OsFTL12* interacts with *GF14b* and *OsFD1* to form the FRC by competing with *Hd3a* for binding [15].

Moreover, several members of the *FT-like* gene family have been identified to regulate flowering time in different plant species. Orthologs of *FTL9* in *B. distachyon* (*BdFTL9*) and maize (*ZmFTL9*) are reported to be involved in flowering [16–18]. Additionally, *FTL10*, the closest paralog of *FTL9*, is involved in flowering control in rice [19–21]. However, a recent study has demonstrated that *FTL9* does not affect flowering in both loss-of-function and gain-of-function experiments and therefore more directly affects grain size and number in rice [22]. OsFTL9 protein from *Nipponbare* (subspecies *japonica*) contained a stop codon in the PEBP domain, predicting its non-functionality [23]. During rice evolution and domestication, the loss-of-function allele of *FTL9* emerged and became fixed, with a non-sense mutation identified in the second exon among *O. rufipogon* accessions, rendering FTL9 as a non-functional protein. The mechanism highlights a strategy that when maternal resources are sufficient, wild plants increase their offspring number and prevent the increase of offspring size by the action of *FTL9*, which helps expand their habitats in a fluctuating environment. Furthermore, it also suggests the functional diversification of *FTL9* and *FTL10* in rice, where *FTL10* regulates flowering like other grasses, while *FTL9* switched its function to regulate grain size and number [22]. As a dietary staple for over half of the global population, rice consistently holds a central position in the realm of plant science. Flowering time regulation in rice is a well-studied pathway involving *FT-*like genes that are particularly important in response to photoperiodic cues. FT-like and its homolog proteins act as a mobile signal that moves from leaves to SAM where it triggers the activation of floral meristem identity genes and initiates flowering. Based on the studies of *Hd3a* and *RFT1*, the functions of *FT-*like genes have been well characterized in flowering induction. However, the involvement of florigens in varied day lengths in flowering control and other agronomic characteristics is largely unclear. Therefore, this study investigates the response of rice *FT-*like genes, *FTL9* and *FTL10*, under SDs. Our results indicated that *ftl10* exhibits redundant roles with other *FTLs* in regulating flowering time in rice under SDs. Critically, overexpression of *FTL10* but not *FTL9* accelerated flowering in rice under SDs which provides further insights into the molecular basis and functional divergence between *FTL10* and *FTL9* in the regulation of flowering in rice.

## Methods

### Plant materials and growth conditions

The *Nipponbare* (*O. sativa* L. ssp. japonica) cultivar seeds provided by the Zhejiang Provincial Key Laboratory of Crop Genetic Resources, college of Agriculture and Biotechnology, Zhejiang University was used as plant material in this study. The procedures used for plant material collection complied with relevant institutional, national, and international guidelines and legislation. Mature seeds were soaked in deionized water and transferred to a net floating on a 0.5mM CaCl_2_ solution. Afterward, seeds were germinated for 2 days at 28 °C in the dark and were transferred to soil. Plants were grown in growth chambers under SD conditions (30 °C 10 h light/25°C 14 h dark) and LD conditions (30 °C 14 h light/25°C 10 h dark). Light was provided by fluorescent white light tubes (700 mmol/m^2^/s). Sampling was done using seedlings or leaves used in the experiments harvested at Zeitgeber 4. All samples (each containing three biological replicates) were immediately frozen in liquid nitrogen and processed or stored at -80 °C for further RNA extraction.

### Vectors construction and plant transformation

To generate *OsFTL9 and OsFTL10* overexpression lines, the full-length coding sequences *OsFTL9 and OsFTL10* were amplified from *Nipponbare* (*O. sativa* L. ssp. japonica) cDNA with the primers set Fw-OsFTL9-EcoR1/Re-OsFTL9-EcoR1 and Fw-OsFTL10-EcoR1/Re-OsFTL10-EcoR1 (Supplementary Table 1), and cloned into the pCAMBIA1390 with the control of the maize (*Zea mays*) *UBIQUITIN* (*UBI*) promoter. *OsFTL10*, *OsHd1, and OsGI* target sequences in sgRNA expression cassettes were cloned into CRISPR-Cas9 system constructs with U3 promoter or U6a promoter to generate mutants. The primers used for vector construction are listed in Supplementary Table 1. Transgenic rice plants were generated by *A. tumefaciens* strain AGL1 using rice embryogenic callus.

### RNA expression analysis

Full-expanded leaves were harvested at the lighting point around 5 weeks under SDs and LDs for gene expression analysis. Total RNA was extracted from leaves using RNAiso Plus (Takara). cDNA was synthesized from 1ug of total RNA with oligo (dT) primers by reverse transcriptase (Promega). The quantitative analysis of gene expression was performed on the Step-One Plus real-time PCR system with the Power Up SYBR Master Mix (ABI) with three biological replicates according to the manufacturer instructions as described [24]. The following cycling conditions were used for Real-time qPCR: 2 min at 95 °C, 40 cycles of 10 s at 95 °C and 40 s at 65 °C, and a final step for melting curve determination (15 s at 95 °C, 1 min at 60 °C and 15 s at 95 °C). GAPDH was used as an internal control. Gene expression was calculated using the 2^−ΔΔCt^ method following [25]. All primers used in RT-qPCR experiments are listed in Supplementary Table 1.

For transcriptome analysis, RNA was extracted from *osftl10* and WT with three biological replicates under SDs. Library preparation and RNA-sequencing were performed at Novogene Bioinformatics Company (Beijing, China) with illumina NovaSeq 6000 platform (PE150). The detailed information for RNA-seq data is presented in Supplementary Table 2. Clean reads were aligned to the Rice genomic sequence (*O. sativa*_323_v7.0) and downloaded from the Phytozome database (Version 12.0) using Hisat2 [26]. Differentially expressed genes were selected with Hochberg-adjusted *P*-values (FDR) < 0.05 and |log2 (Fold change)| >1 setting as the threshold. The GO terms pathways were carried with FDR < 0.05 and were considered as significantly altered and get the GO annotations based on biological process.

### Phylogenetic analysis

The phylogenetic tree was constructed by MEGA software using the Neighbor-Joining method. Alignments of the protein sequences were produced by ClustalW. The percentage of replicate trees in which the associated taxa clustered together in the bootstrap test (1000 replicates) are shown next to the branch. Accession numbers for the Phytozome database website (https://phytozome-next.jgi.doe.gov/) genes referred to phylogenetic trees were as follows: *FT* (AT1G65480), *TSF* (AT4G20370), *TFL1* (AT5G03840), *BdFTL4* (Bradi1g38150), *BdFTL1* (Bradi1g48830), *BdFTL2* (Bradi2g07070), *BdFTL10* (Bradi2g19670), *BdFTL9* (Bradi2g49795), *BdFTL6* (Bradi3g08890), *BdFTL12* (Bradi3g48036), *BdFTL4* (Bradi4g35040), *BdFTL5* (Bradi5g14010), *FTL8* (LOC_Os01g10590), *FTL1* (LOC_Os01g11940), *FTL9* (LOC_Os01g54490), *FTL13* (LOC_Os02g13830), *FTL5* (LOC_Os02g39064), *FTL6* (LOC_Os04g41130), *FTL10* (LOC_Os05g44180), *RFT1* (LOC_Os06g06300), *Hd3a* (LOC_Os06g06320), *FTL12* (LOC_Os06g35940), *FTL4* (LOC_Os09g33850), *FTL11* (LOC_Os11g18870), *FTL7* (LOC_Os12g13030).

### Yeast two-hybrid assays

The full-length coding sequences of *OsFTL9* and *OsFTL10* were amplified from *Nipponbare* cDNA and cloned into pGBKT7. The full-length coding sequences of *OsFD1* and *OsGF14c* were amplified from *Nipponbare* cDNA and cloned into pGADT7. Primers for the constructs are listed in Supplementary Table 1. The recombinant plasmids were co-transformed into yeast strain AH109 using the lithium acetate-based transformation protocol. Positive colonies grown on SD/-Leu/-Trp (-LT) medium were transferred onto selective SD/-Leu/-Trp/-His/-Ade (-LTHA) medium. Positive interactions on -LTHA medium were observed after incubation at 30 °C for approximately 4 days.

### BiFC assays

The full-length coding sequences of *OsFTL9*, *OsFTL10*, *OsFD1*, and *OsGF14c* were amplified and constructed into vectors containing either N- or C-terminal-enhanced yellow fluorescence protein fragments. Gene sequences were amplified by PCR and restriction cloned into the (*Bam* HI and/or *Eco* RI). Primers for the constructs are listed in Supplementary Table 1. The recombinant plasmids were transformed into *A. tumefaciens* strain EHA105, and transiently co-expressed in *N. benthamiana* leaves through *Agrobacterium*-mediated transformation adjusted to OD600 = 0.8. YFP Fluorescence signals were observed after 2–3 days using a confocal laser scanning microscope (Olympus FV3000).

## Results

### Phylogeny of *FT-Like* genes in*A. thaliana*, *B. distachyon*, and *O. sativa*

**Fig. 1:**
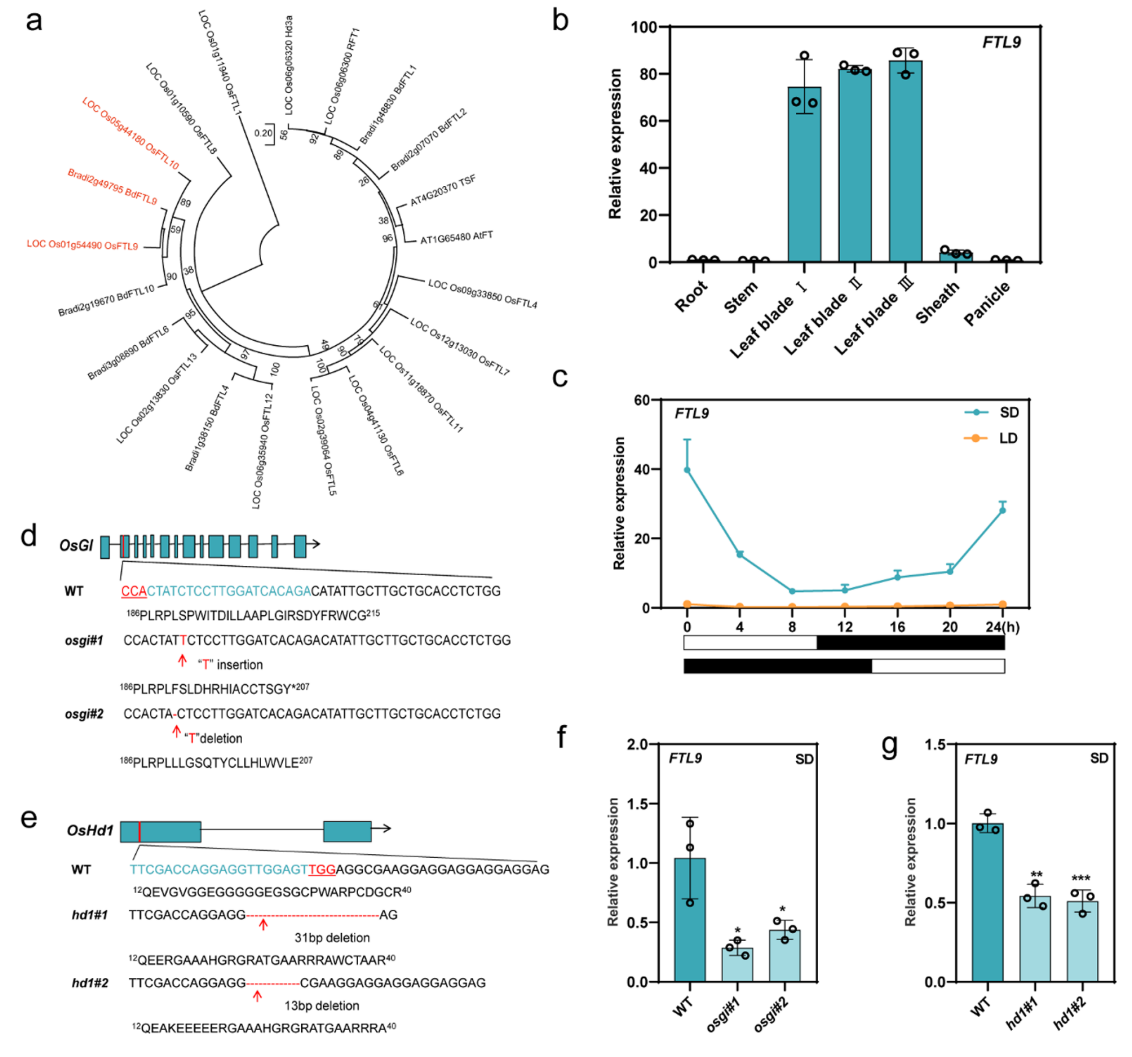
Phylogenetic analysis of FT-like proteins and expression patterns of *FTL-9* in rice. (**a**) A phylogenetic tree of FT-like proteins in *A. thaliana*, *B. distachyon*, and *O. sativa.* The tree was constructed by MEGA software using the Neighbor-Joining method produced by ClustalW with bootstrap test 1000 replicates. The evolutionary distances were computed using the Poisson correction method. (**b**) Expression levels of *FTL9* in different rice tissues. (**c**) Diurnal expression patterns of *FTL9* under LDs and SDs. Abscissas represent the zeitgeber time (ZT) point. The white and black bars parallel to the horizontal axis represent light and dark photoperiods respectively. (**d**) Schematic diagram of *GI* and construction of *gi* knockout mutants using CRISPR-cas9 approach to develop transgenic rice lines. Blue boxes represent exons and black lines represent introns. The sgRNA sequence that specifically targets *GI* is indicated in blue. The PAM sequence is underlined in red. The red dashes indicate the deletion of base pairs and the insert base pairs are marked in red. (**e**) Schematic diagram of *Hd1* and construction of *oshd1* knockout (CRISPR-Cas9) transgenic rice lines. Blue boxes represent exons and black lines represent introns. The sgRNA sequence that specifically targets *Hd1* is indicated in blue. The PAM sequence is underlined in red. The red dashes indicate the deletion of base pairs, and the insert base pairs are marked in red. (**f**) qRT-PCR analysis shows the relative expression levels of *FTL9* in WT and *gi cas9* lines under SDs. (**g**) qRT-PCR analysis of *FTL9* relative expression levels in WT and *hd1 cas9* lines under SDs. Leaf samples were harvested from plants grown for 5 weeks. Data are means ± standard deviation. Asterisks indicate the *P*-value calculated using ANOVA: ***: *P* < 0.001; **: *P* < 0.01; *: *P* < 0.05; ns = not significant

Flowering time control is a critical agronomic trait that determines the fate of crop production. Various flowering genes, including *FT*, *GI*, and *HD*, orchestrate the flowering activation complex to regulate flowering time. Unlike the two FT-like protein homologs in * A. thaliana*, there are 13 FT orthologs in rice (*O. sativa*) and 6 FT-like orthologs in *B. distachyon* [17, 27, 28]. Due to the close sequence alignment of *OsFTL9* and *OsFTL10* with *BdFTL9*, which has strongly influenced the flowering transition when overexpressed in *B. distachyon* [17], we asked whether *FTL9* and *FTL10* can influence the flowering transition in rice. Therefore, we aligned the protein sequences of *FTs* in *A. thaliana*, * B. distachyon*, and rice and found a similarity, illustrating that *B. distachyon* and rice *FTs* share the same protein sequences and will function in flowering control (Fig. 1a). Thus, we further investigated the biological significance and molecular basis of *FTL9* and *FTL10* in rice by investigating them with CRISPR-cas9 and OE lines to study them in detail.

### Tissue-specific and diurnal expression pattern of *FTL9* in rice

Tissue-specific expression of a gene is also very important which allows efficient utilization of resources and adaptation to diverse conditions. To study the tissue-specific expression pattern of *FTL9*, we performed qRT-PCR to study the relative expression patterns of *FTL9* in different plant tissues. We observed significantly higher *FTL9* transcription in leaves (Fig. 1b). Further, we noticed that in leaf blade III, the expression of *FTL9* was higher compared to other leaf blades, indicating that *FTL9* accumulates in leaves, specifically in leaf blade III.

In *B. distachyon*, *FTL9* is a short-day-specific gene [17, 29]. Since *FT* genes usually have diurnal expression patterns, we examined the oscillation of *FTL9* every 4 h during the day-night cycle. Accordingly, we observed a high relative expression of *FTL9* at the end of the dark and low expressions in light time when plants were grown in SDs. However, the relative expression of *FTL9* was significantly repressed at any time point when plants were grown in LDs (Fig. 1c). Altogether, these results indicated the tissue-specific behavior of *FTL9* specifically induced under SDs in rice.

### *GI* and *Hd1* positively regulate *FTL9* expression in rice under SDs

Flowering initiation is a complex agronomic trait that defines the transition between vegetative and reproductive stages and is controlled by various flowering-related genes and internal cues, such as circadian rhythm and photoperiod. Many factors are involved in the control of photoperiodic flowering, including genes like *GIGANTEA* (*GI*) and *Heading date* 1 (*Hd1*) [30, 31]. *GI* plays a central role in photoperiodic flowering, both dependent and independent of the gene *CONSTANS* (*CO*), the key gene in the photoperiod pathway that triggers FT in leaves [32]. Therefore, on the first note, we prepared *gi* loss-of-function mutants with the help of CRISPR-cas9 technique (Fig. 1d).

Similarly, *Hd1*, the *CO* ortholog in rice, plays a dual role in flowering control, promoting flowering under SDs through the expression of *Hd3a*, the *FT* ortholog, and suppressing flowering under LDs by the repression of *Hd3a* [33]. Therefore, we prepared *hd1* mutants using CRISPR-Cas9 (Fig. 1e).

Since the accumulation of these flowering-related genes in SDs and their role in flowering time control, we observed that the relative expression of *FTL9* in *GI* loss-of-function was significantly reduced, indicating the involvement of *GI* in the expression regulation of *FTL9* (Fig. 1f). Interestingly, when we measured the expression of *FTL9* in *HD1* loss-of-function under SDs, we observed that the expression of *FTL9* was obviously decreased compared with WT (Fig. 1g), suggesting that *hd1* positively regulates the expression of FTL9 in rice under SDs.

### Tissue-specific and diurnal expression pattern of *FTL10* in rice

**Fig. 2:**
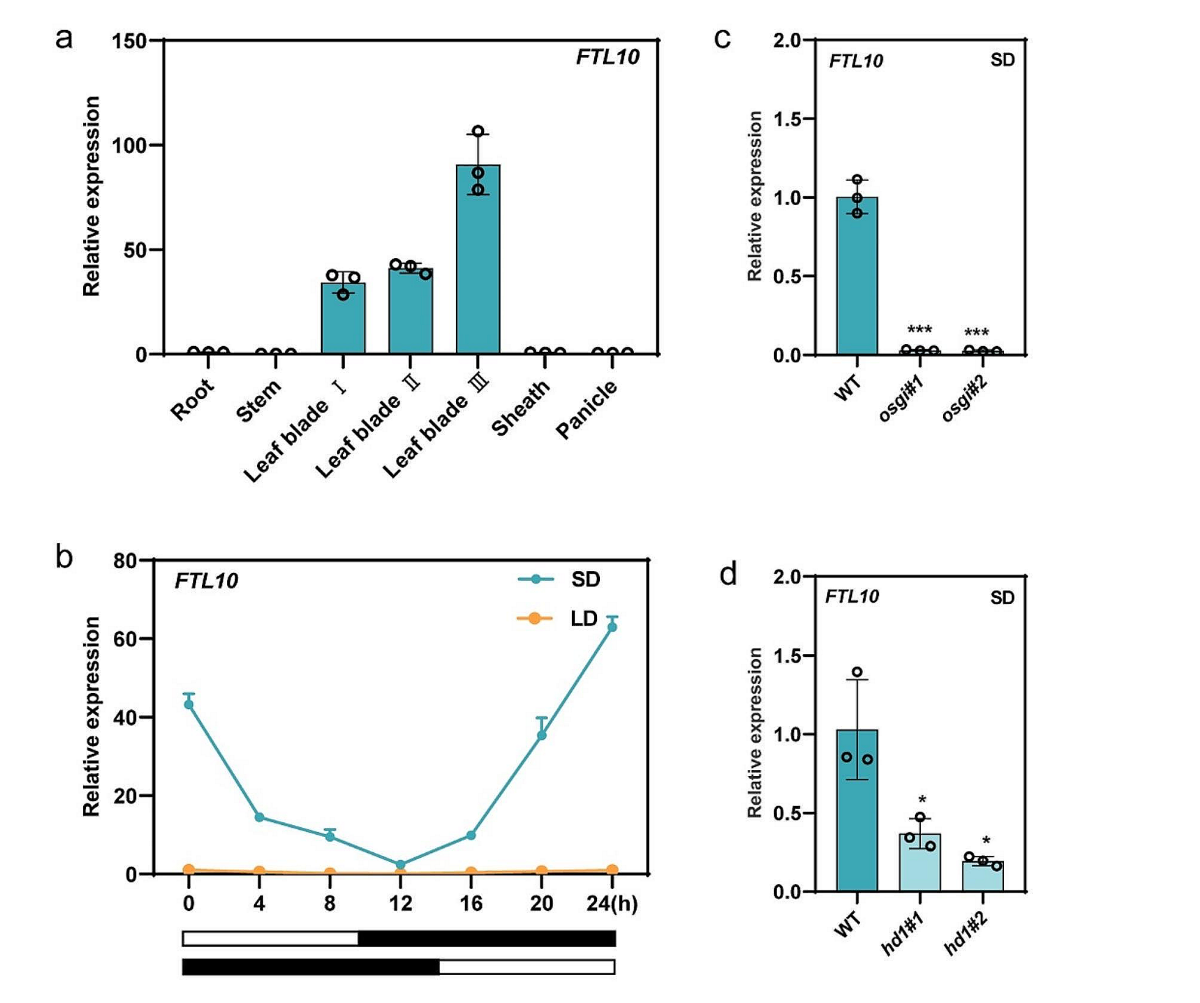
Expression pattern of *FTL10* in different tissues and day lengths. (**a**) Expression levels of *FTL10* in different rice tissues. (**b**) Diurnal expression patterns of *FTL10* under LDs and SDs. Abscissas represent the zeitgeber time (ZT) point. The white and black bars parallel to the horizontal axis represent light and dark periods, respectively. (**c**) qRT-PCR analysis shows the relative expression levels of *FTL10* in WT and *osgi cas9* lines under SDs. (**d**) qRT-PCR analysis of *FTL10* relative expression levels in WT and *hd1 cas9* lines under SDs. Samples were harvested from plants grown for 5 weeks. Data are means ± standard deviation. Asterisks indicate the *P*-value calculated using ANOVA: ***: *P* < 0.001; **: *P* < 0.01; *: *P* < 0.05; ns = not significant

To study the tissue-specific expression pattern of *FTL10*, we performed qRT-PCR of *FTL10* in different tissues of rice. We observed significantly higher expression of *FTL10* accumulation in leaves (Fig. 2a). Further, we noticed that in leaf blade III, the expression of *FTL10* was higher compared to other leaf blades, following the same trend as observed in *FTL9*, indicating that *FTL10* accumulates in leaves, specifically leaf blade III.

Since *FT* genes usually have diurnal expression patterns, we examined the oscillation of *FTL10* every 4 h during the day-night cycle. Accordingly, we observed a high relative expression of *FTL10* at the end of the dark and low expressions in light time when plants were grown in SDs. However, the relative expression of *FTL10* was significantly repressed at any time point when plants were grown in LDs (Fig. 2b). Altogether, these results indicated the tissue-specific behavior of *FTL10* specifically induced under SDs in rice.

### *GI* and *Hd1* positively regulate *FTL10* expression in rice under SDs

Furthermore, since a higher accumulation of *FTL10* in rice under SDs, we observed that the relative expression of *FTL10* in *gi* loss-of-function was significantly reduced, indicating the involvement of *GI* in the expression regulation of *FTL10* (Fig. 2c). Interestingly, when we measured the expression of *FTL10* in *HD1* loss-of-function under SDs, we observed that the expression of *FTL10* was obviously decreased compared with WT (Fig. 2d), suggesting that *HD1* positively regulates the *FTL10* in rice under SDs.

### Overlapping functions of *OsFTL10* in flowering control in rice

**Fig. 3:**
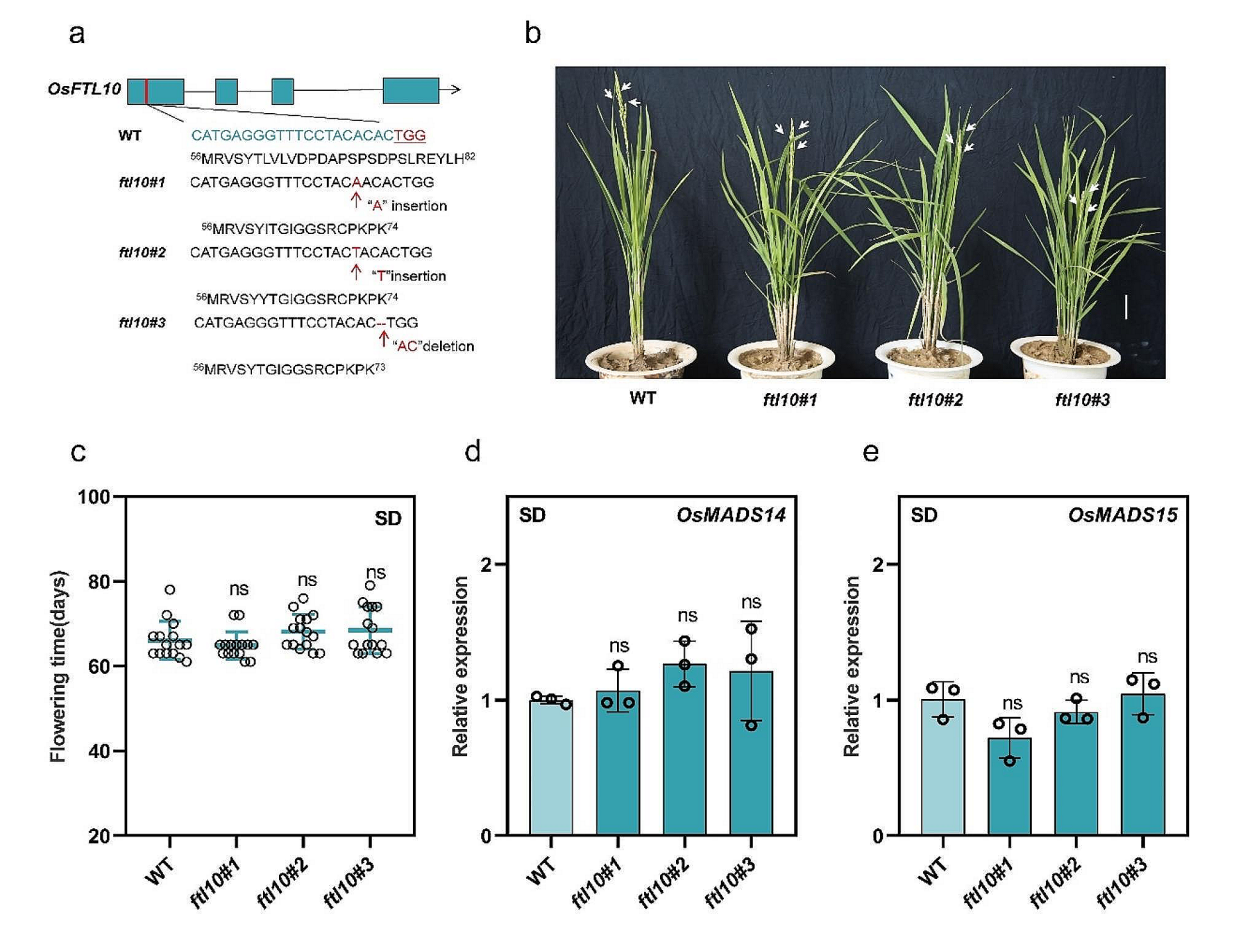
Regulatory roles of *ftl10* loss-of-function in flowering time control in rice under SDs. (**a**) Schematic diagram of *FTL10* and construction of *ftl10* knockout transgenic rice lines using CRISPR-Cas9. Blue boxes represent exons and black lines represent introns. The sgRNA sequence that specifically targets *FTL10* is indicated in blue. The PAM sequence is underlined in red. The red dashes indicate the deletion of base pairs and the inserted base pairs are marked in red. (**b**) Representative flowering phenotypes of WT and *ftl10* under SDs. White arrows point to spikes. Scale bars, 4 cm. (**c**) Measurement of days to flowering time in WT and *osftl10* under SDs. (*n* = 15 plants). (**d**) and (**e**) qRT-PCR analysis of *MADS14* and *MADS15* expression in WT and *ftl10* lines under SDs. Data are means ± standard deviation. Asterisks indicate the *P*-value calculated using ANOVA: ***: *P* < 0.001; **: *P* < 0.01; *: *P* < 0.05; ns = not significant

Since, OsFTL9 from *Nipponbare* (subspecies japonica) is predicted to be non-functional and does not affect flowering in both loss-of-function and gain-of-function experiments [22, 23], we asked whether *FTL10* can perform any role in flowering time control in rice. Therefore, we generated *ftl10* loss-of-function mutants using the CRISPR/Cas9 approach to analyze the *FTL10* deficiency on flowering in rice (Fig. 3a). However, we observed no significant phenotypic differences and days to heading between the WT and *FTL10*, indicating that *FTL10* has redundant roles with other FTL genes in the biological control of flowering in rice (Fig. 3b, and c). Furthermore, the relative expression levels of *MADS14* and *MADS15* observed in *ftl10* showed no significant differences between WT and *ftl10* loss-of-function, indicating FTL10 has no influence over flowering control in rice under SDs (Fig. 3d and e).

### Transcriptome profiling and agronomic characterization of FTL10 loss-of-function

**Fig. 4:**
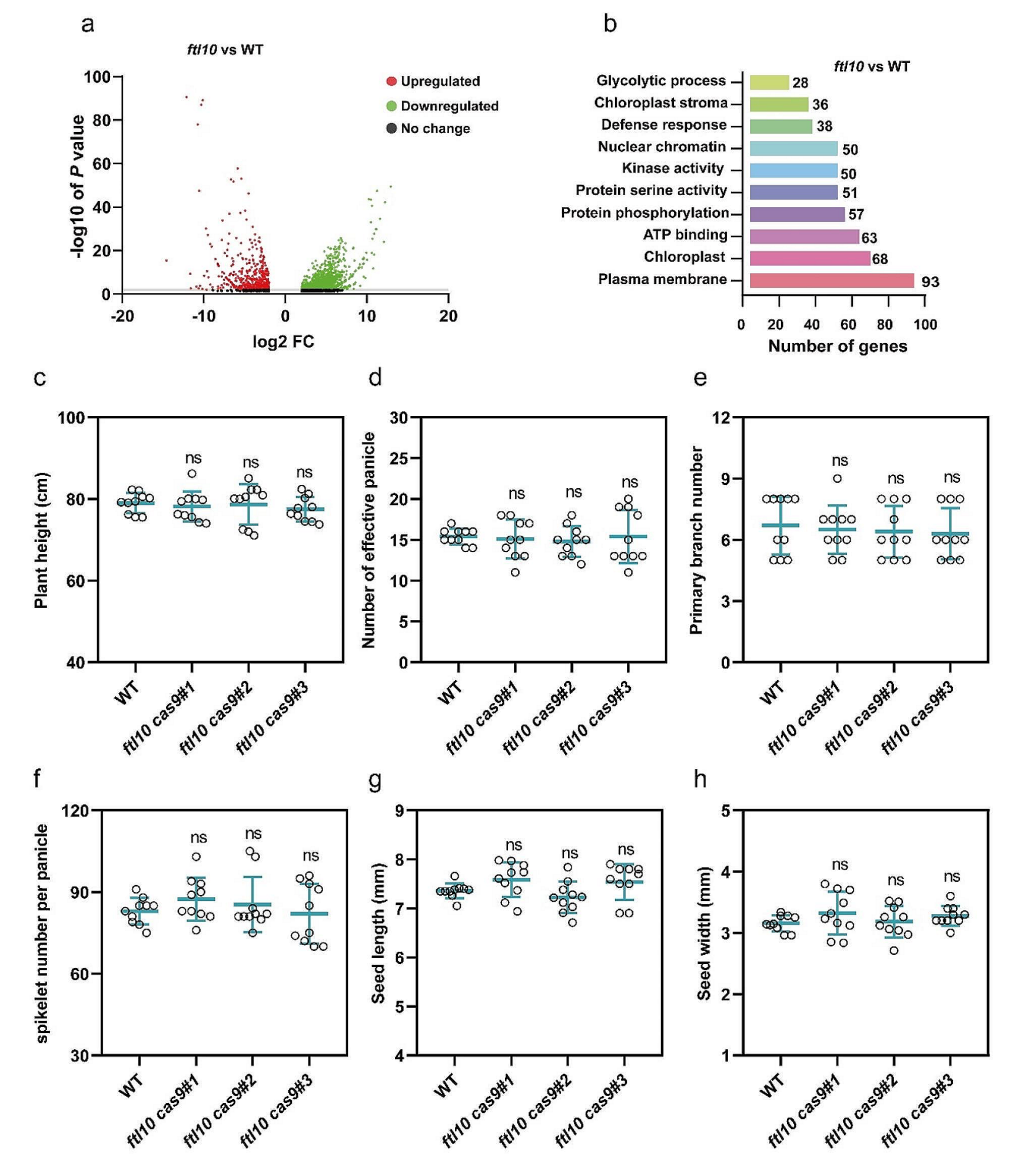
Regulatory roles of *ftl10* loss-of-function in flowering time control in rice under SDs. (**a**) Volcano plot of significant up-and down-regulated differentially expressed genes in *osftl10* vs. WT in rice leaves under SDs. (**b**) Gene Ontology (GO) enrichment analysis for differentially expressed genes in *osftl10* compared with WT in rice leaves under SDs. (**c**) Measurement of plant height of WT and *osftl10* under SDs (*n* = 10 plants). **(d, e, and f)** Measurement of the number of effective panicles per plant, the number of primary branches per panicle, and spikelets per panicle (*n* = 10 plants). (**g and h**) Measurement of seed length and seed width (mm) among WT and *osftl10* (*n* = 10 plants). Data are means ± standard deviation. Asterisks indicate the *P*-value calculated using ANOVA: ***: *P* < 0.001; **: *P* < 0.01; *: *P* < 0.05; ns = not significant

Transcriptome sequencing of the *FTL10* loss-of-function showed that a significant number of genes and pathways were differentially regulated. Several genes related to plasma membrane and protein kinase activity were differentially expressed among *ftl10* and WT. A higher number of differentially expressed genes were observed for the plasma membrane, chloroplast regulation, and ATP binding followed by protein phosphorylation, protein serine activity, and kinase activity (Fig. 4a and b). However, similar to the phenotypic characterization of *ftl10* in flowering control, our transcriptome analysis did not observe any significant changes in the gene expression profile. Since no noticeable changes were observed in the flowering phenotype, we shifted our observations to agronomic traits to explore any potential alterations in other aspects of plant growth and development. However, we failed to observe any significant changes in plant height, the number of effective panicles, the number of primary branches per panicle, and the number of spikelets per panicle (Fig. 4c, d, e, and f). We also compared seed length and seed width of *ftl10* with WT, however, we did not observe any noticeable changes in seed measurements (Fig. 4g and h), indicating that *FTL10*, although it belongs to a flowering control gene family, does not influence flowering control or other agronomic characterization in rice under SDs.

### Over-expression of FTL10 leads to early flowering in rice under SDs

**Fig. 5:**
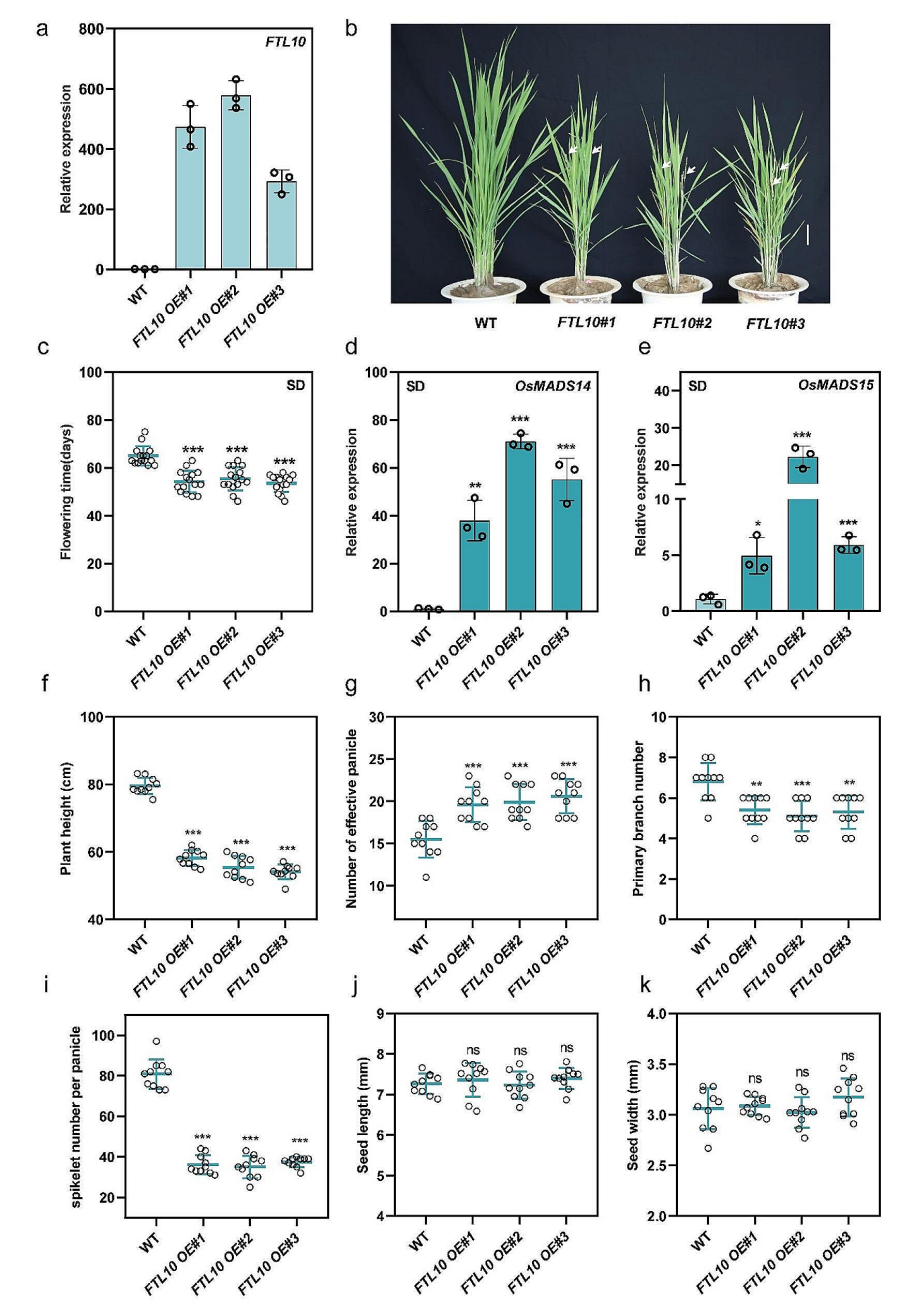
Over-expression of *FTL10* leads to early flowering in rice under SDs. (**a**) qRT-PCR analysis of *FTL10* expression in WT and *FTL10-OE* lines in rice leaves. (**b**) Representative flowering phenotypes of WT and *FTL10-OE* lines under SDs. White arrows point to spikes. Scale bars, 4 cm. (**c**) Measurement of days to flowering in WT and *FTL10-OE* lines under SDs. (*n* = 15 plants). (**d**) and (**e**) qRT-PCR analysis of *MADS14* and *MADS15* expression in WT and *FTL10-OE* lines under SDs. Data are means ± standard deviation. (**f**) Measurement of plant height (cm) of WT and *FTL10-OE* lines under SDs (*n* = 10 plants). **(g, h, and i)** Measurements of the number of effective panicles per plant, the number of primary branches per panicle, and spikelets per panicle (*n* = 10 plants). (**j and k**) Measurements of seed length and seed width (mm) among WT and *FTL10-OE* (*n* = 10 plants). Asterisks indicate the *P*-value calculated using ANOVA: ***: *P* < 0.001; **: *P* < 0.01; *: *P* < 0.05; ns = not significant

Next, we observed the overexpressed lines of *FTL10* in rice under SDs. Interestingly, we witnessed a 300-600-fold increase in the relative expression level of *FTL10* overexpression (*FTL10-OE*) in several independent rice lines (Fig. 5a). Moreover, we observed significant differences in the phenotype of WT and *FTL10-OE* plants under SDs. There were significant differences in plant height between *FTL10-OE* and WT, indicating *FTL10* to be an ideal growth-regulating gene in rice (Fig. 5b). More interestingly, there was a highly significant difference in time to flowering between WT and *FTL10-OE* plants under SDs. *FTL10-OE* plants flowered approximately 9.8 days earlier than their respective WT (Fig. 5c). Furthermore, we also observed the relative expression of *MADS14* and *MADS15* in *FTL10-OE* to check their involvement in flowering time control in rice under SDs. Most interestingly, we found a highly significant relative expression level of *MADS14* and *MADS15* in *FTL10-OE* compared with WT (Fig. 5d and e). The relative expression level of *FTL10* was 35–70 folds higher in *FTL10-OE* compared with WT; however, a 2-15-fold increase was observed for *MADS14* and *MADS15* in *FTL10-OE* compared to WT. Considering agronomic traits other than flowering, we observed significant differences in plant height, the number of effective panicles per plant, the number of primary branches per panicle, and the number of spikelets per panicle among *FTL10-OE* and WT (Fig. 5f, g, h, and i). *FTL10-OE* plants exhibited a significantly higher number of effective panicles with a significant reduction in primary branches and spikelets per panicle. However, there were no observable changes noticed in seed length and seed width among *FTL10-OE* and WT (Fig. 5j and k), maybe following the mechanism that when maternal resources are sufficient, wild plants increase their offspring number and prevent the increase of offspring to help expand their habitats in a fluctuating environment. Altogether, these results suggested that *FTL10*, when overexpressed in rice, can influence the relative expression levels of *MADS14* and *MADS15*, alternatively playing a significant role in flowering time regulation and other agronomic traits in rice under SDs. However, further investigation is warranted to elucidate the roles of *FTL10* in regulating rice agronomy and development.

### Over-expression of FTL9 has negligible effects on flowering and agronomic traits in rice under SDs

**Fig. 6:**
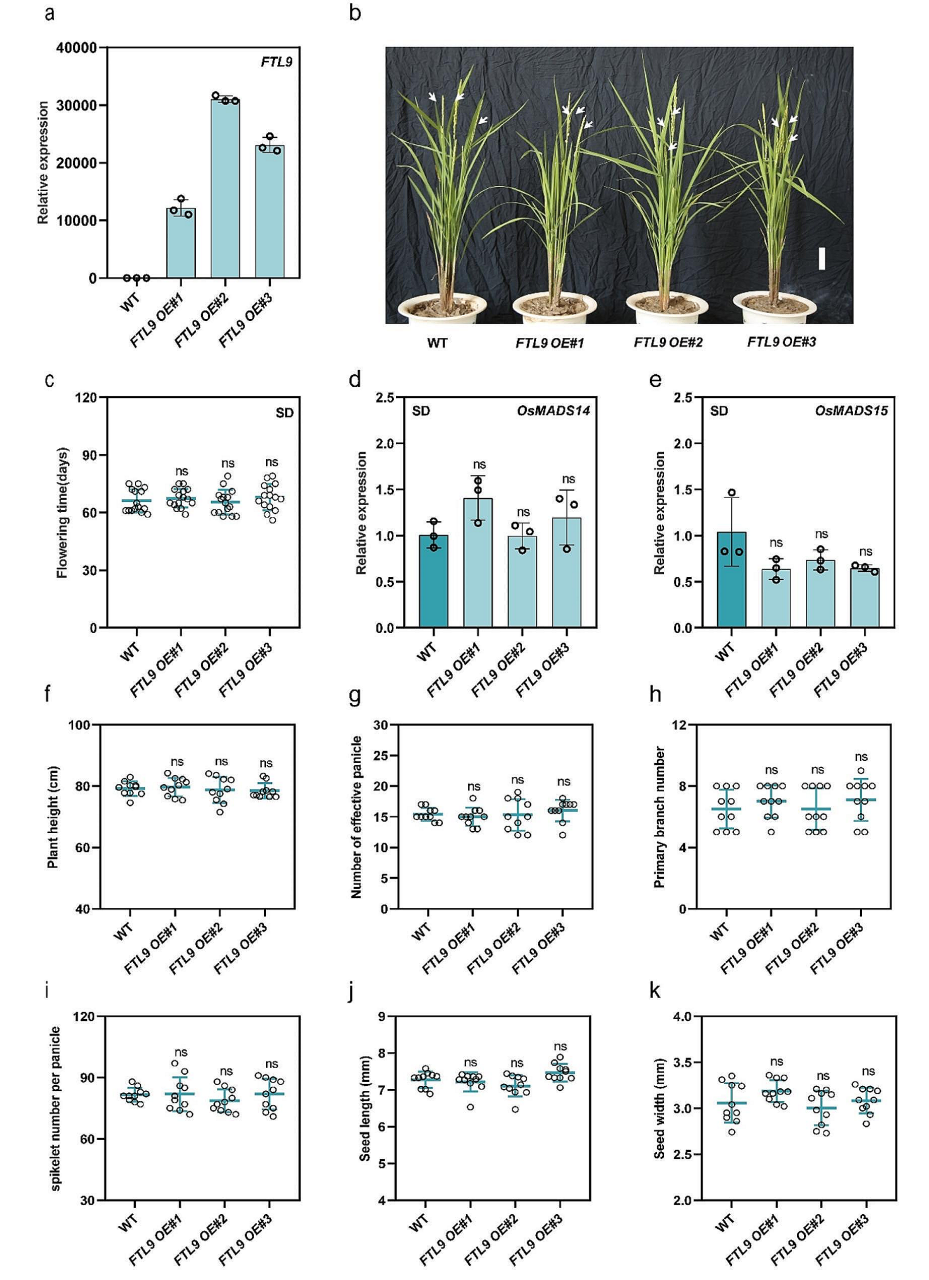
Over-expression of *FTL9* in rice under SDs. (**a**) Relative expression of *FTL9* in WT and *FTL9-OE* lines in leaves. (**b**) Representative flowering phenotypes of WT and *FTL9-OE* lines under SDs. White arrows point to spikes. Scale bars, 4 cm. (**c**) Days to flowering in WT and *OsFTL9-OE* lines under SDs. (*n* = 15 plants). (**d**) and (**e**) qRT-PCR analysis of *MADS14* and *MADS15* expression in WT and *OsFTL9-OE* lines under SDs in rice. (**f**) Measurement of plant height (cm) of WT and *FTL9-OE* lines under SDs (*n* = 10 plants). **(g, h, and i)** Measurements of the number of effective panicles per plant, the number of primary branches per panicle, and spikelets per panicle (*n* = 10 plants). (**j and k**) Measurements of seed length and seed width (mm) among WT and *FTL9-OE* (*n* = 10 plants). Data are means ± standard deviation. Asterisks indicate the *P*-value calculated using ANOVA: ***: *P* < 0.001; **: *P* < 0.01; *: *P* < 0.05; ns = not significant

Considering the regulatory roles of *FTL10* in flowering control in rice, we investigated whether overexpression of *FTL9* could influence rice development. Therefore, we constructed overexpressed lines of SD-induced FTL9 (*FTL9-OE*) in rice. The relative expression level of *FTL9* was highly increased, ranging from 10k to 30k-fold in our several independent *FTL9-OE* lines (Fig. 6a). However, it was very surprising to observe no significant differences in the phenotype and days to flowering between the WT and *FTL9-OE* plants under SDs (Fig. 6b and c). Therefore, we also observed the relative expression of *MADS14* and *MADS15* in *FTL9-OE*. We found negligible variations in the expression patterns of *MADS14* and *MADS15* in *FTL9-OE* compared with WT (Fig. 6d and e), suggesting that *FTL9*, when overexpressed in rice, exerts no discernible influence on flowering regulation in rice. Since we did not observe any changes in flowering, we investigated other agronomic traits to explore any potential alterations in rice growth and development. However, there were no observable changes in plant height, the number of effective panicles, the number of primary branches per panicle, and the number of spikelets per panicle among *FTL9-OE* and WT (Fig. 6f, g, h, and i). We also compared seed length and seed width of *FTL9-OE* with WT, however, we did not observe any noticeable changes in seed measurements (Fig. 4j and k), indicating that *FTL9-OE* does not exert any changes in flowering control or other agronomic characteristics in rice under SDs.

### The mechanism of FTL9 and FTL10 in the flowering control in rice

**Fig. 7:**
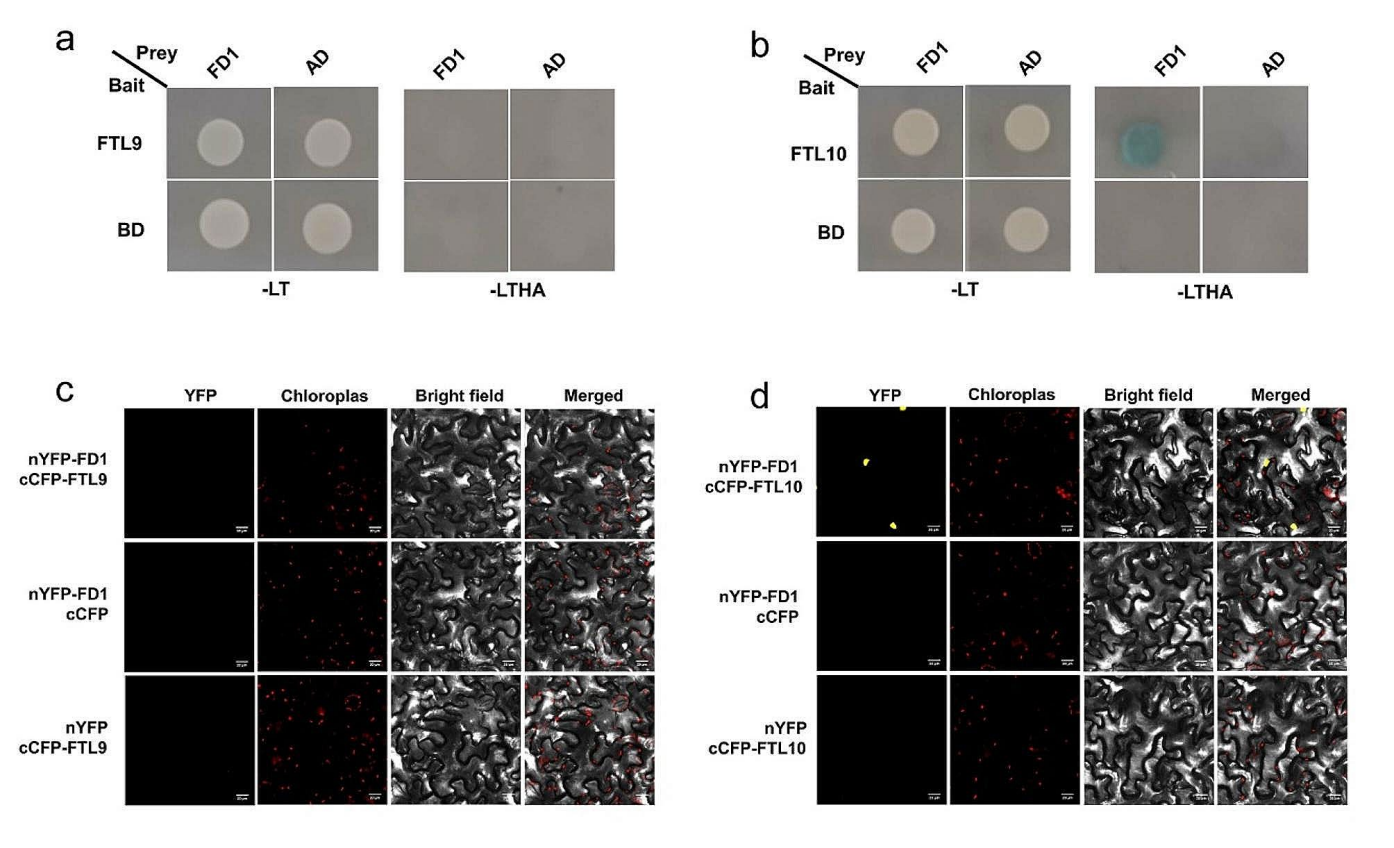
Interactions of *FTL9* and *FTL10* with *FD1* in yeast and tobacco. (**a**) and (**b**) Interaction between FTL9/FTL10 and FD1 proteins in yeast cells. Yeast cells grown on selective media without Leu, Trp (-LT), His and Ade (-LTHA) represent positive interactions. (**c**) and (**d**) Bimolecular fluorescence complementation (BiFC) assay of the interactions between FTL9/FTL10 and FD1 in *N. benthamiana* leaves. Scale bar, 20 μm. N-YFP and C-YFP clones containing empty vectors were used as negative controls

Flowering initiation is a complex process that requires the interaction of several candidate genes involved in the activation of the flowering. Therefore, to check the interaction of *FTL9* and *FTL10* with other flowering candidate genes, we performed yeast two-hybrid (Y2H) and bimolecular fluorescence complementation (BiFC). Our assay for Y2H between FTL9 and FD1 showed no interaction, while there was a strong interaction noticed between FTL10 and FD1 (Fig. 7a and b). This conspicuous difference in interaction patterns elucidates that FTL9 may not possess direct implications to regulate flowering. However, FTL10 can interact with other candidates to activate the flowering complex for flowering initiation. Furthermore, to confirm our results through BiFC assay, there was still no interaction between FTL9 and FD1, while there was a solid interaction noticed between FTL10 and FD1 (Fig. 7c and d), depicting that FTL10 and FD1 can interact with other flowering genes to activate the flowering complex in rice. We expanded our investigation to GF14c to observe its regulatory roles in the flowering pathway in rice and found no interaction between FTL9 and GF14c (Supplementary Fig. 1), indicating that FTL9 may have different regulatory mechanisms independent of GF14c. However, there was a strong interaction observed between FTL10 and GF14c (Supplementary Fig. 1), suggesting that FTL10 may interact with GF14c to regulate flowering in rice, indicating a direct functional relationship between these two genes in the flowering pathway. Altogether, these results elucidate that, unlike FTL9, the strong interactive signals observed in Y2H and BiFC assays between FTL10, FD1, and GF14c suggest that FTL10, might be directly involved in the flowering control pathway. These findings highlight the specific molecular interactions in shaping the dynamics of flowering time regulation in rice.

## Discussion

Flowering initiation is a complex developmental process that ensures the reproductive success of plants, critically influenced by environmental cues. Day length influences the expression levels of several flowering-related genes that interact in the flowering initiation network. Under LD plants, *CONSTANS* (*CO*) interact with the *cis*-element of *Flowering Locus T* (*FT*) to induce flowering [34] illuminates how flowering is induced under LDs; however, how SD plants trigger flowering is still unclear. Therefore, in the present study, we characterized two SD-induced *FTs*, *FTL9* and *FTL10*, to investigate their role in flowering time control in rice under SDs. FT family proteins play central roles in regulating reproductive development in the flowering control pathway, although several FT homologs repress the flowering transition [35]. The biological significance of *FT*-like genes is predominant to induce flowering in SD plants. In SD conditions, the loss of function of *ftl9* can lead to delayed flowering in *B. distachyon*. Conversely, with the transition to long photoperiods in spring, winter plants initiate flowering through various mechanisms, including *FT1* activation and *FTL9* reduction [17]. Hence, *FTL9* functions offer valuable insights for SD plants to meet diverse agricultural demands and adapt to climatic conditions. Recent investigations have reported that *FTL9* orthologs in *B. distachyon* and maize and its paralog *FTL10* are involved in flowering [16–21], however, rice *FTL9* does not affect flowering both in gain-of-function and loss-of-function experiments. This investigation further implies the functional diversification of *FTL9* and *FTL10* in rice. Specifically, *FTL10* regulates flowering in rice like other grasses, while *FTL9* shifts its function to regulated grain size and number [22].

Appropriate flowering time in rice is imperative for enhancing environmental adaptability and augmenting grain yield. Photoperiod, a vital environmental signal, governs flowering. The intricate interplay of light stimuli and circadian rhythms orchestrates the regulation of photoperiodic flowering. In rice, *Heading date 1* (*Hd1*), the *CO* ortholog, exerts dual regulation, promoting flowering under SDs by enhancing the *FT* counterpart *Hd3a* and retarding flowering under LDs by suppressing *Hd3a* [33]. A previous study unveiled that *Ghd7* forms a complex with *Hd1*, leading to the suppression of *Hd3a* transcription and concurrent inhibition of *Early Heading date 1* (*Ehd1*) expression, thereby negatively regulating flowering in rice under LDs [36]. Another study identified a mutation in *OsELF3/ELF7*, an Arabidopsis *ELF3* homolog, which markedly elevates *Ghd7* transcription, leading to delayed flowering in rice in both LDs and SDs [37].

Moreover, *Hd1* has contrasting regulatory roles dictated by day length, facilitated by *DAYS TO HEADING 8*, a NUCLEAR FACTOR Y (NF-Y) transcription factor potentially interacting with *Hd1* [38]. Further investigation is warranted to explain the specific pathway involving *CO1* and NF-Y, enabling the activation of *FT1* and the suppression of *FTL9* to promote flowering in rice under SDs. However, despite potential regulation by *CO1*, *FTL9* expression remains relatively unaltered in *CO1* knock-down lines under LDs [17]. Additional investigations are required to discern alternative upstream regulators of *FTL9* in the photoperiodic flowering pathway.

Ehd1 facilitates flowering in rice under both SDs and LDs. It plays a pivotal role in two SD modules, *OsGI-OsCO-Ehd1-Hd3a* and *OsGI-OsMADS15-Ehd1-Hd3a*, where *Ehd1* acts upstream of *Hd3a*, directly upregulating *Hd3a* expression to promote flowering. Enhanced *OsFTL10* overexpression, coupled with elevated *Ehd1* and *Hd3a* levels in transgenic plants, is likely to accelerate flowering in rice [39]. Given that *Ehd1* plays a pivotal role in integrating flowering signals from both SDs and LDs, its regulation by various genes, including the feedback from *FTL10*, merits further investigation in rice. In the context of SDs, *MADS51*, downstream of *GI*, functions as a flowering promoter. Reduction in the expression level of *MADS51* in *FTL10*-*OE* lines contradicts the early flowering phenotype, potentially attributed to the elevated *Ehd1* expression downstream of *MADS51* [19]; however, *RFT1* displayed a modest downregulation. Therefore, it is suspected that *FTL10* or other genes related to flowering may have modulated the expression of *MADS51*, analogous to the effects observed with *Hd3a* and *RFT1*, where, in response to RNAi-induced *Hd3a* downregulation, *RFT1* was activated to promote flowering under SDs [8]. *RFT1* primarily initiates flowering under LDs, whereas *Hd3a* predominantly promotes flowering under SDs. *Hd3a* and *RFT1* respond to photoperiodic regulation by undergoing transcription, as evidenced by their silencing via RNAi, leading to a complete absence of flowering even 300 days post-sowing [7, 40].

In Arabidopsis, *FD* with FD PARALOG (FDP) is redundant. Double mutations of *fd/fdp* flower later than *fd* single mutations, and almost completely suppress precocious flowering of 35 S: FT [41]. In addition, by mutating *FD3* and *FD4* in rice, floral-promoting factors were identified. Mutations in *fd4*, but not in *fd3*, can delay flowering and activation of *MADS* targets. However, flowering was not delayed as much as in *fd1* mutants, indicating a less prominent role compared with *FD1*. Also, *fd1*/*fd4* double mutants did not further delay flowering compared with *fd1* single mutants, proposing that *FD1* activated *FD4* transcription in rice [42]. Thus, this arrangement does not fully exclude redundancy but suggests a complex regulatory network.

Further, *MADS62* controls pollen maturation and germination, partially redundantly with *MADS63* and *MADS68* in rice [43]. *FD1* and *FD4* promoted *OsMADS62* expression, a function they probably perform after meristem commitment, during inflorescence and flower development. It is also proposed that FAC containing FD1 directly targets the relative expression of *MADS15* [44]. However, neither FD1 nor FD4 is bound to the MADS14, MADS15 or MADS34/PAP2 promoter in rice [42]. Other proteins could possibly stabilize FD4 and FD1 in vivo and allow binding to these loci. Alternatively, the rice FD1-*MADS* connection might be indirect. Data in support of a direct connection have not been as thoroughly repeated and validated in rice as they have been in Arabidopsis, in which direct contacts between FD and the C-box element in the AP1 promoter were disproved [45–48]. Further assessment of in vivo binding is necessary.

Thus far, no *FT*-like gene with dual functionality in both SD and LD contexts has been identified. Knockout mutants of *FTL10* displayed no significant phenotypic alterations under SD conditions in rice. Genetic redundancy in which the effect of mutations in a single gene is strongly enhanced by a second mutation in a related gene has been demonstrated in *osfd1-2* and *osfd4-1*, which do not have completely overlapping functions [42]. However, the overexpression of *FTL10*, an *Hd3a* homolog, induced early flowering in rice. A study demonstrated that the overexpression of both genes *FTL10* and *Hd3a*, under the same promoter, yielded varying flowering periods, potentially attributed to amino acid variations between the two proteins [19].

Based on the study, several observations were made differentiating between *FTL10* and *Hd3a*: (1) *FTL10* exhibited high expression in young leaves, whereas *Hd3a* primarily expressed in mature leaves; (2) Both FTL10 and Hd3a proteins were localized in the cytoplasm and nucleus, yet BiFC assay results indicated that FTL10 predominantly interacted with GF14c in the nucleus, while Hd3a interacted with GF14b in the cytoplasm; (3) *FTL10-OE* led to increased *Ehd1* expression, which, in turn, may affect the flowering pathway. The relative expression level of *MADS51* was also decreased in *FTL10* transgenic plants, suggesting that flowering regulation of *FTL10* may exhibit subtle distinctions from the pivotal florigens *Hd3a* and *RFT1* [19].

According to our observations, the expression patterns of *MADS14* and *MADS15* in *FTL9-OE* lines showed no significant differences when compared with WT. However, *MADS14* and *MADS15* expression in *FTL10-OE* showed highly significant differences between the *OE* lines and WT, suggesting that *FTL10*, when overexpressed in rice under SDs, can play significant roles in flowering control. Moreover, we also demonstrated in the current study that *FTL10* can interact with *FD1* to induce floral transition in rice. *FTL10* interacts with *FD1* in Y2H and BiFC assays; however, it is not clear whether the interaction of *FTL10* and *FD1* requires the presence of 14-3-3 or not. Therefore, further investigation is needed to demonstrate the interaction of 14-3-3 with *FT*-like genes to regulate flowering time in rice under SDs.

## Conclusion

In conclusion, this study investigates the functional diversity of *FTL9* and *FTL10* in rice flowering time regulation under SD conditions, giving rise to a nuanced perspective. While there is a non-sense mutation identified in the second exon of *FTL9* among *O. rufipogon* accessions [22], rendering *FTL9* as a non-functional protein, *FTL10*, the closest homolog of *FTL9*, exerted no discernible influence over flowering time regulation in rice under SDs. Nevertheless, overexpression of *FTL10*, but not *FTL9*, substantially accelerated flowering, and regulated other agronomic traits without regulating seed size. Moreover, the augmented expression patterns of *MADS14* and *MADS15* in *FTL10* overexpression lines proposed an alternative regulatory pathway in flowering regulation in rice under SDs. Collectively, these findings imply the existence of unexplored *FT*-like genes involved in flowering time regulation in rice under varying environmental cues, encouraging further exploration to study the comprehensive orchestration of flowering time control in rice across diverse environmental settings.

### Electronic supplementary material

Below is the link to the electronic supplementary material.

Supplementary Material 1

Supplementary Material 2

Supplementary Material 3: Supplementary FiguresSupplementary Fig. 1: Interactions of *FTL9* and *FTL10* with GF14c

## Data Availability

All data supporting the findings of this study are available within the paper and within its supplementary materials.

## Abbreviations

FAC: Flowering activation complex
FRC: Florigen repression complex
FTL9: Flowering Locus T-like 9
FTL10: Flowering Locus T-like 10
CO: Constans
SD: Short-day
LD: Long-day
FD1: Ferredoxin
Hd1: Heading date 1
Hd3a: Heading date 3a
RFT1: Rice flowering locus T1
OE: Over-expression
GI: Gigantea
Hd1: Heading date 1
WT: Wild type
Y2H: Yeast two hybrid assay
BiFC: Bimolecular fluorescence complementation
MFT-like: Mother of ft and tfl1-like
TFL1-like: Terminal flower1-like
DEGs: Differentially expressed genes
Ehd1: Early heading date 1
