# Supplementary figures and images for: Functional divergence of FTL9 and FTL10 in flowering control in rice
Source: BMC Genomics. 2024 Jun 5;25:562. doi: 10.1186/s12864-024-10441-9 (PMC11151565; doi:10.1186/s12864-024-10441-9)

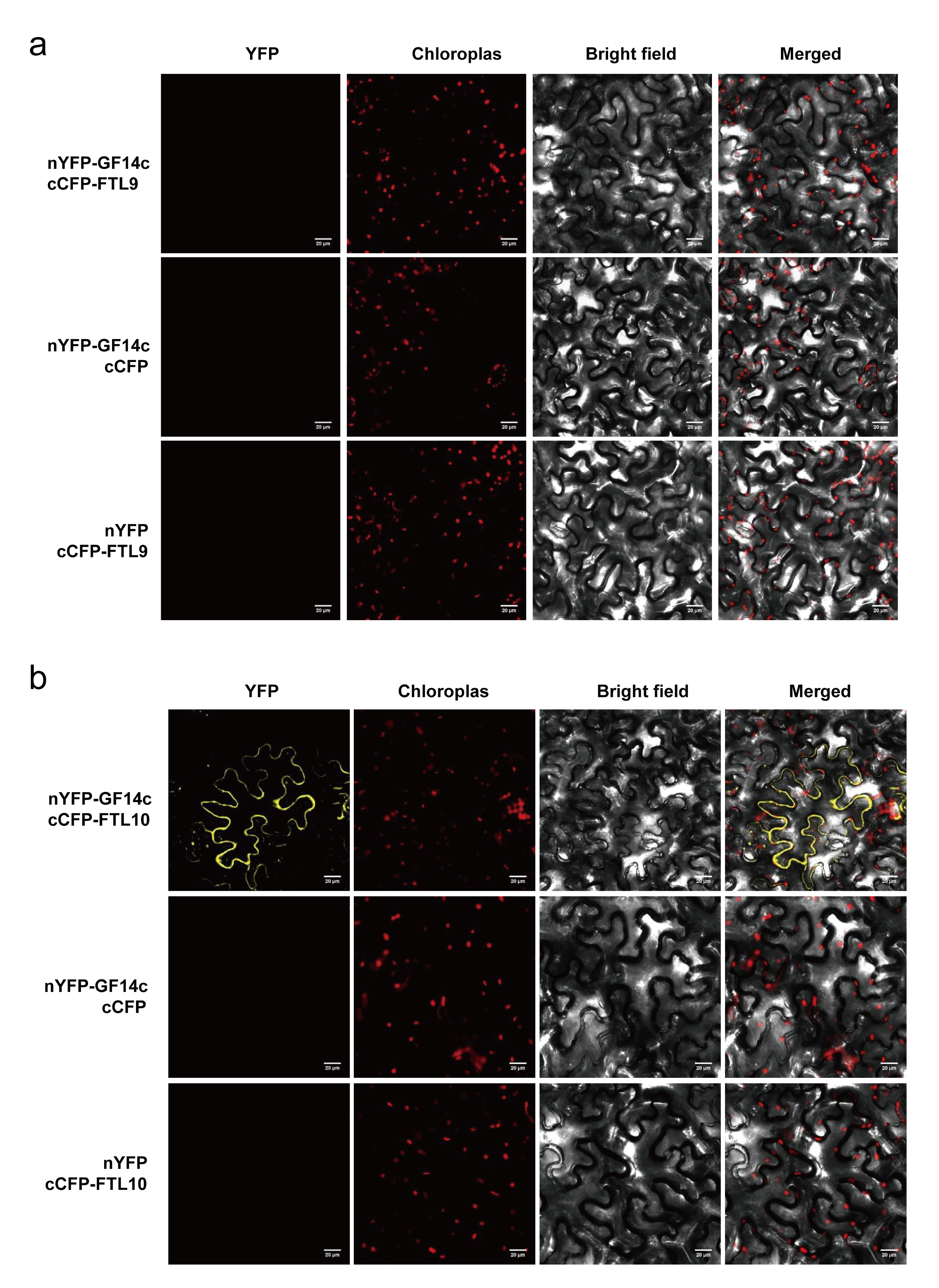

Supplement: Supplementary file 3 — Supplementary Material 3: Supplementary FiguresSupplementary Fig. 1: Interactions of FTL9 and FTL10 with GF14c [file 12864_2024_10441_MOESM3_ESM.png]
